# The Effect of Triptolide-Loaded Exosomes on the Proliferation and Apoptosis of Human Ovarian Cancer SKOV3 Cells

**DOI:** 10.1155/2019/2595801

**Published:** 2019-05-21

**Authors:** Huan Liu, Ming Shen, De Zhao, Dan Ru, Yourong Duan, Chenhuan Ding, He Li

**Affiliations:** ^1^Traditional Chinese Medicine Department, Renji Hospital, School of Medicine, Shanghai Jiao Tong University, Shanghai 200127, China; ^2^State Key Laboratory of Oncogenes and Related Genes, Shanghai Cancer Institute, Renji Hospital, School of Medicine, Shanghai Jiao Tong University, Shanghai 200032, China

## Abstract

Triptolide has been proven to possess anticancer efficacy; however, its application in the clinical practice was limited by poor water solubility, hepatotoxicity, and nephrotoxicity. In this study, a triptolide-loaded exosomes delivery system (TP-Exos) was constructed and its effects on the proliferation and apoptosis of SKOV3 cells* in vitro *and* in vivo *were observed. SKOV3-exosomes (SK-Exos) were collected by ultracentrifugation and ultrafiltration centrifugation. TP-Exos was constructed by sonication and ultrafiltration centrifugation. SK-Exos and TP-Exos were characterized by transmission electron microscopy, western blotting, nanoparticle-tracking analysis, and high-performance liquid chromatography. Cellular uptake of exosomes, 3-(4,5-dimethylthiazol-2-yl)-2,5-diphenyltetrazolium bromide (MTT) assay, bromodeoxyuridine (BrdU) cell proliferation assay, and cell apoptosis experiment were used to study the effect of TP-Exos on ovarian cancer in vitro. Tumor-targeting study of exosomes, monitoring the tumor volume of mice, and TdT-mediated dUTP Nick-End labeling (TUNEL) assay were used to evaluate the effect of TP-Exos on ovarian cancer in vivo. The toxicity of TP-Exos in vivo was evaluated by liver and kidney function and histopathology of major organs (heart, liver, spleen, lung, kidney, and ovary). The results revealed that TP-Exos not only have the general characteristics of exosomes but also have high drug encapsulation efficiency. Besides, PKH26 labeled exosomes (PKH26-Exos) could be uptaken by SKOV3 cells, and Dir labeled exosomes (Dir-Exos) could be enriched to the tumor site of tumor bearing mice. Furthermore, the cytotoxic and apoptotic effects on SKOV3 cells of TP-Exos were weaker than those of free TP, and tumor cell proliferation inhibition and tumor growth inhibition were stronger than that of free TP. Moreover, TP-Exos have toxic effect on liver and spleen. In conclusion, the TP-Exos could be a promising strategy for ovarian cancer, but they need to be further optimized to attenuate the damage to liver and spleen.

## 1. Introduction

Triptolide (TP) is a major active ingredient of the Chinese herbal medicine* Tripterygium wilfordii *Hook.f. It has been reported that TP has not only immunosuppressive and anti-inflammatory effects, but also an antitumor activity, which can inhibit the proliferation of various types of cancer cells* in vitro* and reduce the growth and metastasis of some solid tumors* in vivo *[1–6]. It has been found that TP has a great potential against ovarian cancer and could inhibit the proliferation of such cells and induce their apoptosis [7–10]. Unfortunately, some studies have revealed that TP not only has poor solubility but also could cause severe reproductive-system toxicity [11, 12], hepatotoxicity [13–15], and nephrotoxicity [16, 17]. Generally, the problems mentioned above hinder the potential clinical applications of TP to ovarian cancer treatment.

In recent years, controlled-release delivery systems and targeted drug delivery systems have become some of the most promising innovations in the field of drug delivery, for example, liposomes and polymersome micelles, which are used to increase drug solubility, improve drug bioavailability, and to reduce toxicity (adverse effects) of drugs [18]. In our previous studies, two types of TP delivery systems, TP-loaded micelles [19] and triptolide-loaded liposomes-Ca/P nanoparticles (TP@Lips-Ca/P) [20], have been constructed. The results revealed that they can enhance the antitumor effect and reduce the toxicity of TP. Nonetheless, the TP-loaded micelles are material of xenobiotic origin and are easily cleared by the mononuclear phagocyte system (MPS, which is mainly composed of monocytes and macrophages). Regarding TP@Lips-Ca/p, although the uptake by MPS was reduced by polyethylene glycol (PEG), the interaction between target cells and barrier cells was also reduced. Because of the deficiencies in the drug delivery system of two types of nanoparticles, we continuously seek innovative nanosized materials of biological origin to further promote the use of TP in anti–ovarian cancer therapy.

Exosomes are natural nanosized vesicles (30 ~ 150 nm) secreted by a variety of cells, and also a promising drug delivery carrier. Exosomes as drug delivery carrier have many advantages, for example, a small particle size, biocompatibility, biological barrier penetration, a phospholipid bilayer similar to that of liposomes, low toxicity, and low immunogenicity [21–23]. At present, exosomes derived from B cells, dendritic cells, T cells, macrophages, mesenchymal stem cells, and tumor cells are often employed as drug delivery carriers [24–28]. Exosomes have already been tested for the delivery of different antitumor agents, such as curcumin [29, 30], paclitaxel [31, 32], and doxorubicin [25, 33]. These exosomes can deliver drugs to recipient cells and alter their function. At present, there are few studies on the treatment of ovarian cancer with drug-loaded exosomes. In this study, we prepared TP-Exos and investigated the effects of TP-Exos on the ovarian cancer cell line SKOV3* in vitro* and* in vivo*.

## 2. Materials and Methods

### 2.1. Reagents

Dulbecco's modified Eagle's medium (DMEM), fetal bovine serum (FBS), and a penicillin/streptomycin solution were purchased from Gibco® (Invitrogen, USA). The BCA Protein Assay Kit, Hematoxylin and Eosin Staining Kit, and SDS-PAGE Preparation kit were purchased from Beyotime (Shanghai, China). TP was bought from the Chengdu Must Bio-technology and FITC Annexin V Apoptosis Detection Kit I, In Situ Cell Death Detection Kit, and BD Pharmingen™ BrdU Flow Kit from BD Bioscience (Shanghai, China). Acetonitrile and 10% formalin neutral buffer solution were purchased from Sangon Biotech (Shanghai) Co., Ltd. 1,1′-dioctadecyl-3,3,3′,3′-tetramethylindotricarbocyanine iodide (Dir), PKH26 Red Fluorescent Cell Linker Kit, dimethyl sulfoxide (DMSO), 4,6-diamidino-2-phenylindole (DAPI), trypsin, phosphate buffered saline (PBS), and MTT from Sigma-Aldrich (Shanghai, China), whereas a horseradish peroxidase- (HRP-) conjugated anti-rabbit IgG antibody and anti-CD9 and anti-CD81 monoclonal antibodies were acquired from Abcam (Cambridge, UK). The female Balb/c nude mice (4 ~ 6 weeks old) were purchased from the Shanghai Renji Hospital Animal Facility.

### 2.2. Cell Culture

The human ovarian cancer cell lines SKOV3 and A2780 were acquired from the National Laboratory for Ontogenesis and Related Genes, Cancer Institute of Shanghai Jiao Tong University, and were cultured in DMEM, which was supplemented with 10% of FBS and 1% of the penicillin/streptomycin solution. All cells were maintained at 5% CO_2_ and 37°C.

### 2.3. Isolation of Exosomes

Isolation of exosomes was performed according to the literature [34] with some modifications. To avoid contamination by the FBS-derived exosomes, FBS was centrifuged at 110,000 ×* g* for 6 h to remove exosomes before the experiment. The human ovarian cancer SKOV3 cells were cultured in DMEM, which was supplemented with 10% of exosome-free FBS and 1% of the penicillin/streptomycin solution. The culture supernatant from SKOV3 cells grown in a T75 flask was harvested at 48 h, and exosomes were isolated by ultracentrifugation and ultrafiltration centrifugation. Briefly, the culture supernatants were cleared of cell debris and large vesicles by sequential centrifugation at 300 ×* g* for 10 min, 1000 ×* g* for 20 min, and 10,000 ×* g* for 30 min, followed by filtration using 0.22 *μ*m syringe filters. Then, the sample was centrifuged at 110,000 ×* g* for 6 h to obtain an exosomal pellet. The collected exosomes were washed twice with PBS by means of an Aicon Ultra-15 Centrifugal Filter (10 kDa, Millipore), then passed through a 0.22 *μ*m syringe filter for sterility, and stored at –80°C until use for studies. All procedures were carried out at 4°C.

### 2.4. Loading of Exosomes

Preparation of TP-Exos was conducted according to the literature [26] with some modifications. Namely, 3 mg (protein concentration) of purified exosomes dispersed in 900 *μ*l PBS (pH 7.2 - 7.4) was mixed with 100 *μ*g TP dissolved in 100 *μ*l DMSO, and then the mixture was sonicated by an ultrasonic cell crusher (JY92-II, China) (20% power, 15 cycles of a 2 s pulse/2 s pause). After sonication, the mixture was incubated at 37°C for 60 min to allow for recovery of the exosomal membrane. Excess free TP and DMSO were separated from the mixture by centrifugation at 12,000 ×* g* for 30 min using the Amicon Ultra-0.5 Centrifugal Filter (10 kDa, Millipore) and then washed twice with precooled PBS. Finally, the pellet was suspended in PBS, passed through a 0.22 *μ*m syringe filter for sterility, and stored at –80°C until use for studies.

### 2.5. Characterization of Exosomes

The morphology of SK-Exos and TP-Exos was examined by transmission electron microscopy (TEM). Firstly, the 200 mesh formvar copper grids were exposed to the Glow Discharge technique for 1 min and purified exosomes were fixed with 2% Paraformaldehyde for 5 min. And then, 5 *μ*l of exosomes suspension solution was added onto the grids and incubated for 1 min at 4°C. Subsequently, the grids were negatively stained with 1% uranyl acetate for 2 min, washed with distilled water, and dried out in darkness for 10 min under room temperature. Finally, the samples were analyzed by TEM (Tecnai G2 Spirit TWIN) at 80 Kv.

Nanoparticle-tracking analysis (NTA) was performed to analyze the size distribution of SK-Exos and TP-Exos according to the literature [35, 36]. Firstly, the exosomes samples were diluted in PBS (1:10 dilution). And then, three 30 s videos were recorded. Finally, particle size was analyzed by NTA software (version 3.2, NanoSight).

Western blotting (WB) was carried out to detect the expression of tetraspanins, such as CD9 and CD81, on the surface of exosomes. Protein was extracted from SKOV3 cells, SK-Exos, and TP-Exos using a cell lysis buffer and the total protein concentrations of three samples were determined using the BCA Protein Assay Kit according to manufacturer's recommendations. Then, 5 × loading Buffer was added to the protein samples. After boiling 10 min, 20 *μ*g of proteins was electrophoresed and transferred to a polyvinylidene difluoride membrane. Furthermore, the membranes were blocked with 5% (w/v) skim milk powder and then treated with a primary CD9 and CD81 antibody (1:1000 dilution). Finally, the membranes were incubated with a secondary antibody (1:1000 dilution) and the protein bands were visualized by Western Lightening Chemiluminescence reagents (Millipore).

The concentration of TP in the TP-Exos was determined by high-performance liquid chromatography (HPLC) (Agilent Technologies 1200 series). The HPLC profiles of TP standard solution, SK-Exos solution, and TP-Exos solution were detected by HPLC to investigate the interference of SK-Exos solution on the determination of TP. A series of TP solutions were prepared, and 20 *μ*l samples solution was injected for chromatographic analysis. The standard curve was drawn with the concentration of TP as the transverse coordinate and the average peak area as the longitudinal coordinate. 50 *μ*l of TP-Exos solution was added to 1950 *μ*l acetonitrile to extract the drug and precipitate the exosomal proteins. The precipitated proteins were separated by centrifugation (12,000 ×g for 10 min), supernatant was separated, and 20 *μ*l supernatant samples were analyzed on HPLC system using a reversed-phase column (Zorbax SB-C18, 150×4.6 mm, 5*μ*m). The mobile phase consisted of a mixture of acetonitrile-water (40/60, v/v), and the flow rate was 1.0 ml/min. The column effluent was detected at 218 nm and the column temperature was 30°C. Entrapment efficiency (%) was calculated via the formula: (Drug entrapped/Total amount of drug) × 100%.

### 2.6. Exosome Labeling

Purified exosomes were incubated with 4 *μ*l PKH26 for 15 min at 37°C. Unbound dye was removed by centrifugation at 12,000 ×* g* for 30 min by means of the Amicon Ultra-0.5 Centrifugal Filter (10 kDa, Millipore), and then the pellet was washed twice with precooled PBS. The obtained PKH26-Exos were resuspended in precooled PBS and stored at −80°C protected from light prior to use.

### 2.7. Cellular Uptake of Exosomes

SKOV3 and A2780 cells were incubated with PKH26-Exos (20 *μ*g/ml protein concentration) at 37°C and 5% CO_2_ for various periods. After each time point, the medium was removed, and the cells were washed 3 times with PBS and then stained with 1 ml DAPI (2.5 *μ*g/ml) for 5 min. Subsequently, the cells were fixed with 1 ml 4% paraformaldehyde for 10 min. Internalization of PKH26-Exos by SKOV3 and A2780 cells was observed by fluorescence microscopy (Olympus IX51, Japan). The fluorescence intensity of PKH26 was measured with Image J Software.

### 2.8. A Cytotoxicity Assay

Cytotoxicity of free TP and TP-Exos was measured by an MTT assay. SKOV3 cells were seeded onto 96-well plates at a density of 5 ×10^3^ cells per well 24 h prior to drug treatment. Various concentrations of free TP or TP-Exos were added to cells for 24 or 48 h incubation. Subsequently, free TP- and TP-Exo-containing media were removed, and the cells were incubated at 37°C for 4 h with an MTT solution (0.5 mg/ml). Finally, MTT solution were removed and 150 *μ*l DMSO solution per well was added to dissolve formazan crystals. Formazan concentration was determined by measuring absorbance at 490 nm on an ELISA microplate reader (Bio TEK ELX800, USA), and the number of living cells was calculated from formazan optical density at 490 nm (OD_490_) values.

### 2.9. A Proliferation Assay

The influence of SK-Exos, free TP, and TP-Exos on the proliferation of SKOV3 cells was evaluated by BrdU/DNA double-parameter flow cytometry. According to the results of MTT assay, the concentrations of TP in free TP and TP-Exos groups was 10 ng/ml. The protein concentration in SK-Exos group was the same as the SK-Exos protein concentration in 10 ng/ml TP-Exos group. The protein concentrations of SK-Exos in 10 ng/ml TP-Exos was determined using the BCA Protein Assay Kit according to manufacturer's recommendations. Free TP-, TP-Exo–, and SK-Exo– containing media were added to SKOV3 cells for 24 or 48 h incubation. After treatment, cells were collected and processed according to the respective manufacturers' instructions for the BD Pharmingen™ BrdU Flow Kit. The samples were analyzed on a BD flow cytometer (BD FACSCelesta™, USA). The data were analysed with FlowJo 7.6 Software. The cell proliferation index (PI) was used to indicate the proliferation state of the cell population: PI = (S + G2/M)/(G0/G1 + S + G2/M) × 100%, where the letters denote a percentage of cells in a respective phase of the cell cycle.

### 2.10. An Apoptosis Assay

The effect of SK-Exos, free TP, and TP-Exos on the apoptosis of SKOV3 cells was assessed by double-staining flow cytometry with propidium iodide (PI) and an annexin V–fluorescein isothiocyanate (FITC) conjugate. The concentration of SK-Exos, free TP and TP-Exos was the same as Proliferation Assay. Free TP-, TP-Exo–, and SK-Exo–containing media were added to SKOV3 cells for 24 or 48 h incubation. After that, the cells were processed according to the respective manufacturers' instructions for the FITC Annexin V Apoptosis Detection Kit I. The samples were analyzed on the BD Flow cytometer (BD FACSCelesta™, USA). The data were analysed with FlowJo 7.6 Software.

### 2.11. Tumor Targeting of Exosomes In Vivo

Preparation of Dir-Exos was conducted according to the literature [24] with some modifications. Purified exosomes were incubated with 25 *μ*g/ml fluorescent lipophilic tracer Dir for 15 min at 37°C. Unbound dye was removed by centrifugation at 12,000 ×* g* for 30 min by means of the Amicon Ultra-0.5 Centrifugal Filter (10 kDa, Millipore), and then the pellet was washed twice with precooled PBS. The obtained Dir-Exos were resuspended in precooled PBS, and the particle concentration of it was measured by NTA. Dir-Exos were diluted in PBS to achieve a particle concentration of 7.5 × 10^10^ per ml.

To ensure the number of tumor-bearing mice, 10 female Balb/c nude mice were subcutaneously injected with SKOV3 cells in 100 *μ*l of PBS (2×10^6^ cells) in the right flank. The tumor size was measured by a Vernier caliper. The tumor volume was calculated as V = (d^2^ × D)/2, where d and D represent the shortest diameter of the tumor and the longest diameter perpendicular to the short diameter in mm, respectively. When the tumor volume reached approximately 500 mm^3^, 6 tumor-bearing mice were randomly divided into two groups (n = 3): Free-Dir group and Dir-Exos group. And then, 200 *μ*l Free-Dir (25*μ*g/ml) and 200 *μ*l Dir-Exos (7.5 × 10^10^ per ml) were administered via peritoneal injection. 4, 8, 24 h, and 48 h after injection, tumor-bearing mice were anesthetized with isoflurance, and then the distribution of Dir was detected by an in vivo imaging system (Night OWL II LB983). After 48 h of treatment, tumor-bearing mice were sacrificed and the main organs, such as heart, liver, spleen, lung, kidney, and tumors, were excised for fluorescence imaging. The data were analysed with the Night OWL Indigo Software.

### 2.12. Antitumor Efficacy of TP-Exos in Tumor-Xenografed Nude Mice

To ensure the number of tumor-bearing mice, 40 female Balb/c nude mice were subcutaneously injected with SKOV3 cells in 100 *μ*l of PBS (2×10^6^ cells) in the right flank. When the tumor volume reached approximately 100 mm^3^, 24 tumor-bearing mice were randomly divided into four groups (n = 6): control group, SK-Exos group, free TP group, and TP-Exos group. The control group was treated with 200 *μ*l PBS. The concentrations of TP in free TP and TP-Exos groups were 0.2 mg/kg. The protein concentration in SK-Exos group was the same as the SK-Exos protein concentration in TP-Exos group.

PBS solution, SK-Exos solution, free TP solution, and TP-Exos solution were injected into the tumor-bearing mice via peritoneal injection. The mice were treated twice a week for 4 weeks and the tumor size was measured once a week for 4 weeks by a Vernier Caliper. The tumor volume was calculated as V = (d^2^ × D)/2, where d and D represent the shortest diameter of the tumor and the longest diameter perpendicular to the short diameter in mm, respectively. After treatment, blood was collected by submandibular vein plexus, and then mice were sacrificed. Subsequently, the tumors and the vital organs (such as heart, liver, spleen, lung, kidney, and ovary) of mice were resected, and a digital camera was used for the imaging of tumor. The tumors and the vital organs were fixed in 10% formalin neutral buffer solution for TUNEL assay and histological analysis.

### 2.13. TUNEL Assay of Tumor Tissue

The tumor tissue was embedded in paraffin and sliced into 5 microns. Apoptotic cells in tumor tissue were determined by the TUNEL method using In Situ Cell Death Detection Kit according to the manufacturer's instructions. Apoptotic cells were observed and photographed under fluorescence microscope. Green fluorescence was positive cell and blue fluorescence was nucleus. TUNEL-positive cells and total cells in paraffin sections of tumor tissue were measured with Image J Software.

### 2.14. Systemic Toxicity Evaluation of TP-Exos

Blood was centrifuged at 1,000 × g for 10 min at 4°C, and then serum was collected and stored at - 20°C until analysis. The levels of blood urea nitrogen (BUN), creatinine (Cr), aspirate aminotransferase (AST), and alanine aminotransferase (ALT) were measured in serum samples.

The vital organs (such as heart, liver, spleen, lung, kidney, and ovary) were embedded in paraffin and sliced into 5 microns. The paraffin sections were stained with Hematoxylin and Eosin Staining Kit according to the manufacturer's instructions and examined with a fluorescent microscope.

### 2.15. Statistical Analysis

For all the experiments, data are presented as the mean ± SD (standard deviation). Tests for significance of differences between the groups were performed by the* t* test (two groups) or one-way analysis of variance (one-way ANOVA)(multiple comparisons) in GraphPad Prism 7.0 (GraphPad Software, USA). Data with P < 0.05 were considered significantly different.

## 3. Results and Discussion

### 3.1. Characterization of SK-Exos and TP-Exos

**Figure 1:**
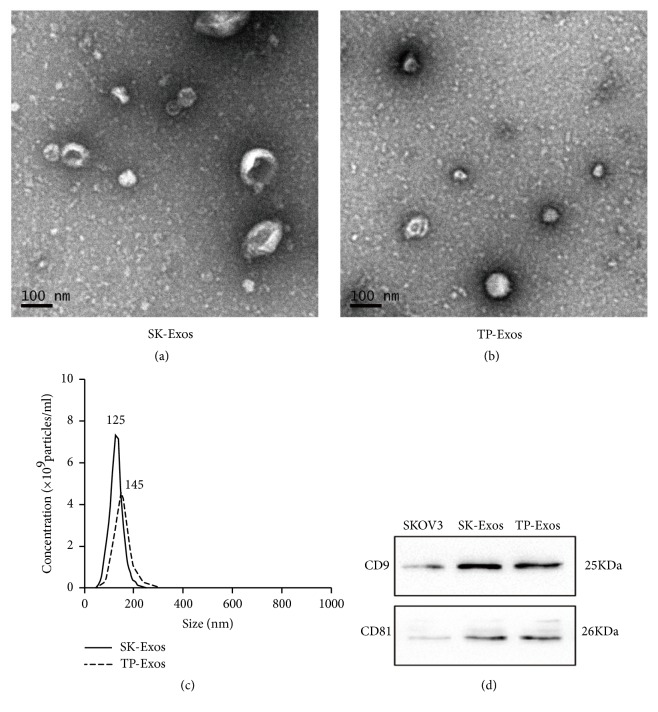
Characterization of SK-Exos and TP-Exos. (a, b) TEM images of SK-Exos and TP-Exos. Scale bar = 100 nm. (c) Size distribution of SK-Exos and TP-Exos measured by NTA. (d) Western blot analysis of SK-Exos and TP-Exos protein expression.

TEM showed that SK-Exos had a typical cup-shaped structure and TP-Exos had an ellipse shape (Figures 1(a) and 1(b)). NTA indicated that the sizes of SK-Exos and TP-Exos were 129.6 ± 1.9 nm and 159.9 ± 2.7 nm, respectively (Figure 1(c)). WB suggested that SK-Exos and TP-Exos were rich in tetraspanins, such as CD9 and CD81 (Figure 1(d)). The encapsulation efficiency of TP in TP-Exos was analyzed by HPLC. The results displayed that the HPLC profiles for TP extracted from TP-Exos and TP reference showed no distinction, indicating SK-Exos solution did not interfere with the determination of TP (Supplementary Figure 2.1). The linear regression equation of TP standard curve was Y = 46.225 X + 32.738, r = 0.9964 (Supplementary Figure 2.2), indicating that the concentration of TP in the range of 0.25 ~ 50 *μ*g/ml had a good linear relationship with its peak area integral value. The encapsulation efficiency of TP in TP-Exos was 76.5 ± 1.8% (Supplementary Table 1). The results of TEM, WB, and NTA showed that SK-Exos had the typical characteristics of exosomes, which indicated that the extraction method of exosomes was feasible. Characterization results of TP-Exos showed that TP-Exos not only had the general characteristics of exosomes but also had high entrapment efficiency of TP, indicating that TP could be loaded into SK-Exos effectively by sonication.

Several methods, such as incubation at room temperature, electroporation, surfactant permeabilization, sonication, hypotonic dialysis, freeze–thaw cycles, extrusion, and cell-mediated packaging, can be utilized to encapsulate a drug in exosomes [23]. Studies have shown that exosomes can increase their drug-loading capacity after sonication. Myung Soo Kim et al. [26] tried different methods to prepare drug-loaded exosomes, and the amount of paclitaxel loaded into exosomes increases in the following order: incubation at room temperature < electroporation < sonication. Matthew J. Haney et al. [37] tested a variety of methods for loading catalase into exosomes, and the amount of catalase loaded into exosomes increased in the following order: incubation at RT < freeze/thaw cycles < sonication ≈ extrusion. According to the above observations, TP was loaded into exosomes by sonication in this study.

After TP was loaded into SK-Exos by sonication, TP-Exos were collected by ultracentrifugation and ultrafiltration centrifugation, respectively. Then, we compared the difference of TP-Exos obtained by two methods by NTA (Supplementary Figure 1(a)). The results showed that the D 10, D 50, and D 90 of TP-Exos collected by two methods had no significant difference, indicating that the two methods have no significant effect on the particle size of TP-Exos (Supplementary Figure 1(b)). D 90 is the corresponding particle size when the cumulative particle size distribution of a sample is up to 90%. Its physical meaning was that the size of particles smaller than 220 nm accounted for 90% of the total number of particles in this study. The particle concentration of TP-Exos in ultracentrifugation group and ultrafiltration centrifugation group was significantly lower than that in SK-Exos group (P < 0.001), and that in ultracentrifugation group was significantly lower than that in ultrafiltration centrifugation group (P < 0.001) (Supplementary Figure 1(c)). The results indicated that there was a loss of exosomes in the process of collecting TP-Exos by the two methods, but the amount of exosomes lost by ultrafiltration centrifugation was lower. Therefore, ultrafiltration centrifugation was used to collect TP-Exos in this study.

In Figure 1, the size of the sample measured by TEM was not consistent with the size of the sample measured by NTA. The reasons for this phenomenon are as follows. TEM can describe the diameter of exosomes and clearly display the structure of exosomes by direct measurement. However, the size distribution data obtained by TEM are often not representative because of the limited scope observed at one time. In addition, the samples of TEM often need to be pretreated by drying, fixation, and freezing, which may affect the size of exosomes. In contrast, NTA can detect the size of exosomes in solution, which provides structural and functional protection for exosomes. NTA allows the exosomes to be measured closer to its original state, which ensures the authenticity and validity of the measured data.

The encapsulation efficiency of TP in TP-Exos was compared with that of two TP delivery systems in our previous study, TP micelles, and TP@Lips-Ca/P nanoparticles [19, 20]. The encapsulation efficiency of TP in TP-micelle, TP@Lips-Ca/P nanoparticles, and TP-Exos was 62.1 ± 3.3%, 72.31 ± 3.11%, and 76.5 ± 1.8%, respectively. The encapsulation efficiency of TP in TP-Exos was similar to that of in TP@Lips-Ca/P nanoparticles. This phenomenon may be related to lipid bilayer structure in exosomes. Exosomes have similar lipid bilayer structure with liposomes [38]. The hydrophobic layer can load the hydrophobic substances, while the inner aqueous cavity can load water-soluble substances. Triptolide is soluble in organic solvents such as ethanol, methanol, dimethylsulfoxide (DMSO), and acetonitrile but not in water. Therefore, through the hydrophobic affinity between the hydrophobic structure of exosomes and the hydrophobic drug, triptolide may be self-assembled into the lipid bilayers. In addition, it has been shown that hydrophobic drug encapsulation efficiency of exosomes was at least two fold higher than for standard liposomes [39]. This may be the reason why the TP-Exos collected by ultrafiltration centrifugation still has a high incorporation efficiency of TP.

### 3.2. Accumulation of SK-Exos in Cells

**Figure 2:**
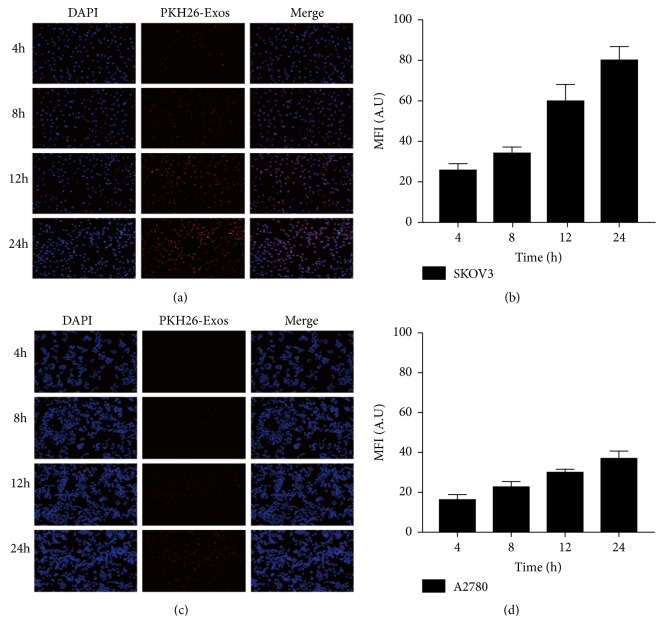
(a, c) Fluorescence microscopy images of exosomes labeled with PKH-26 after incubation with SKOV3 and A2780 cells for 4, 8, 12, or 24 h. Original magnification, 200×. (b, d) Fluorescence intensity statistical analytical diagram of panels (a) and (c). Data are presented as mean ± SD (n = 3).

The ability of exosomes to deliver a drug into target cells is crucial for the therapeutic efficiency of exosomal formulations. We tested whether tumor cells had ability to take up their own exosomes* in vitro*. The results displayed that both SKOV3 and A2780 cells could uptake PKH26-Exos (Figures 2(a) and 2(c)), but accumulation of PKH26-Exos in SKOV3 cells was approximately twice that in A2780 cells (Figures 2(b) and 2(d)). The results suggested that SK-Exos could be taken up by SKOV3 cells, which was similar to the results of Heikki Saari et al. [40], showing that SK-Exos may be able to efficiently deliver a drug to their parental cells.

### 3.3. Cytotoxicity of TP-Exos

**Figure 3:**
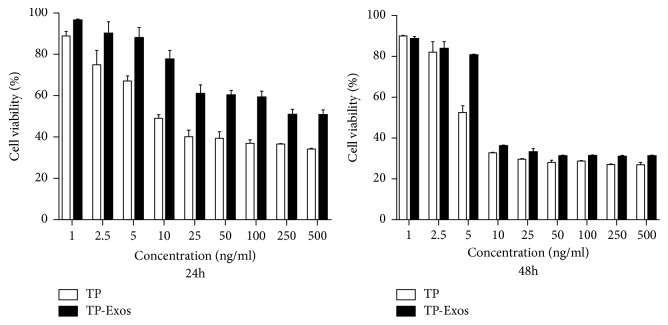
SKOV3 cells were incubated with TP or TP-Exos for 24 and 48 h. The statistical analysis diagram of the results of the MTT assay. Data are presented as mean ± SD (n = 3).

The MTT assay was used to assess the cytotoxicity of different concentration of free TP and TP-Exos on SKOV3 cells after 24 h and 48 h. Statistical analysis of the survival rate of cells was given in Figure 3. After 24 h of treatment, the survival rate of cells in TP-Exos group was significantly higher than that in TP group, and the half-maximal inhibitory concentration (IC50) values of free TP and TP-Exos groups were 25.68 and 270.3 ng/ml, respectively. However, after 48 h, the cell survival rate in TP-Exos group was slightly higher than that in TP group, and the IC50 values of free TP and TP-Exos groups were 10.6 and 18.93 ng/mL, respectively. We can conclude that encapsulation into exosomes may attenuate or delay the cytotoxicity of TP toward SKOV3 cells in vitro, which was similar to the results of Tang K et al. [41].

### 3.4. The Effect of TP-Exos on Proliferation of SKOV3 Cells

**Figure 4:**
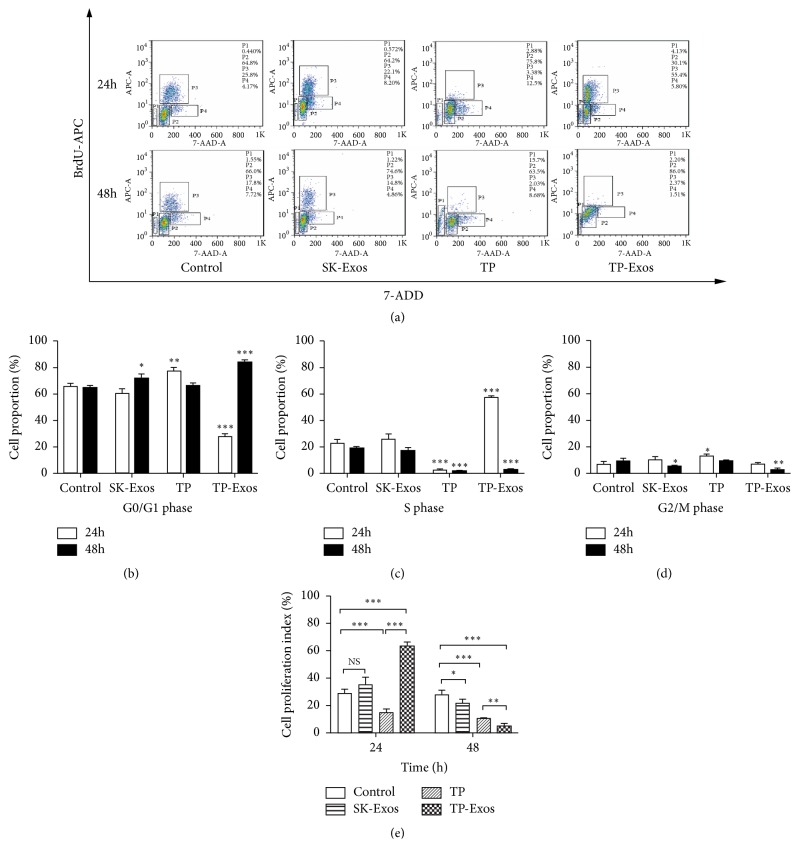
(a) Results of BrdU/DNA double-parameter flow cytometry. P1: cells in sub-G0/G1 phases, P2: cells in G0/G1 phases, P3: cells in S phase, and P4: cells in G2/M phase. (b, c, d) Quantitative statistical analysis of the cell cycle based on panel (a). (e) Statistical analysis of the PI based on panel (a). Data are presented as mean ± SD (n = 3). NS means p > 0.05; *∗*p < 0.05, *∗∗*p < 0.01, and *∗∗∗*p < 0.001.

Exosomes derived from tumor cells may have effects to promote the proliferation of parental cells [42]. Therefore, BrdU/DNA double-parameter flow cytometry was conducted to detect antiproliferative action of TP-Exos toward SKOV3 cells. The results of flow cytometry were shown in Figure 4(a). We first analyzed the effect of SK-Exos, free TP, and TP-Exos on the cell cycle of SKOV3 cells based on Figure 4(a), and the results were shown in Figures 4(b), 4(c), and 4(d). Then, we analyzed the effect of SK-Exos, free TP. and TP-Exos on the PI of SKOV3 cells according to Figures 4(b), 4(c), and 4(d), and the results were shown in Figure 4(e). At 24 h, SK-Exos had no effect on cell cycle, but at 48 h, SK-Exos blocked cells in G0/G1 phase. At 24 h, TP blocked cells in G0/G1 phase, but at 48 h, the number of cells in G0/G1 phase, S phase and G2/M phase was lower than that in control group, indicating that TP induced cell apoptosis. At 24 h, TP-Exos blocked cells in S phase, but at 48 h, TP-Exos blocked cells in G0/G1 phase. After 24 h, the PI of SKOV3 cells treated with SK-Exos was not significantly different from that of the control group (p > 0.05). The PI of the free TP group was lower than that of the control group (P < 0.001); however, the TP-Exos group yielded a greater result than that of the control group (P < 0.001). After 48 h, the PI of SK-Exos group, free TP group, and TP-Exos group was lower than that of the control group (P < 0.05), and the TP-Exos group had a lower PI than did the free TP group (P < 0.01).

In Figure 4(e), PI increased significantly in TP-Exos group at 24 h, which was due to the following reasons. In this study, 10 ng/ml TP-Exos blocked cells in S phase at 24 h and G0/G1 phase at 48 h. S phase arrest may be the reason for the increase of proliferation index in TP-Exos group at 24 h. The arrest of cell cycle by TP-Exos may be related to the concentration of free TP. Wang Y et al. [43] found 12.5 nM/L (4.505 ng/ml) TP could block the cells in S phase at 24 h. Liu L et al. [44] found 10 ng/ml TP could block cells in G0/G1 phase at 24 h. The cell cycle results of the 10ng/ml TP-Exos treated group were consistent with those of Wang et al. at 24 h and were consistent with those of Liu et al at 48 h. These results suggested that TP may be released less from TP-Exos at 24 h and almost the entire after 48 h. Studies have shown that drug-loaded exosomes exhibited a time-dependent release and the cumulative release of drugs were over 50% after 24 h [45] and over 90% at 48 h [46]. Furthermore, exosomes may carry more drugs into the cells, so that the inhibitory effect of TP-Exos on cell proliferation is superior to that of free TP at 48 h.

### 3.5. The Influence of TP-Exos on Apoptosis of SKOV3 Cells

**Figure 5:**
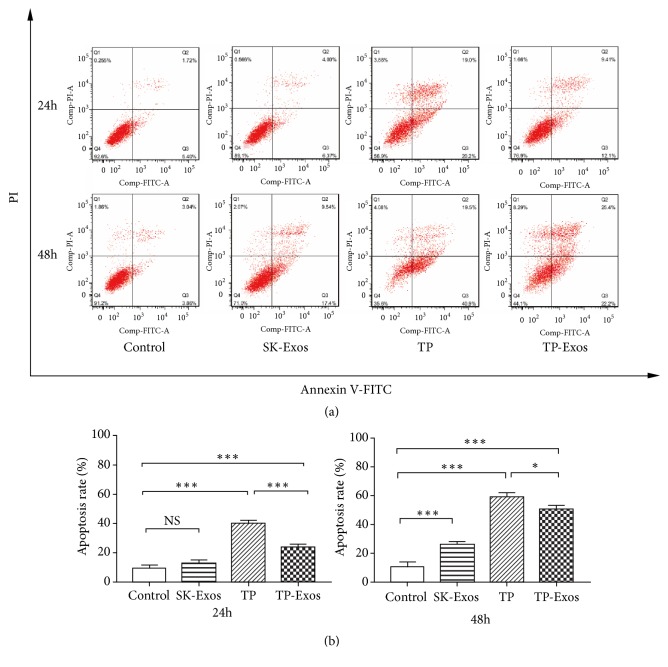
SKOV3 cells were incubated with TP or TP-Exos for 24 and 48 h. (a) Results of PI/annexin V double-staining flow cytometry. (b) Statistical analysis of the apoptosis rate in panel (a). Data are presented as mean ± SD (n = 3). NS means p > 0.05; *∗*p < 0.05, and *∗∗∗*p < 0.001.

Exosomes derived from tumor cells may have effects to promote the apoptosis of parental cells [47]. Therefore, PI/Annexin V–FITC double staining flow cytometry was conducted to detect the effect of TP-Exos on the apoptosis of SKOV3 cells. The results were illustrated in Figure 5. After 24 h of treatment, the apoptosis rate of the SK-Exos group was not significantly different from that of the control group (P > 0.05). The percentages of apoptotic cells in the free TP group and TP-Exos group were higher than this parameter of the control group (P < 0.05). After 48 h of treatment, the apoptotic rates of the SK-Exos group, free TP group, and TP-Exos group were significantly higher relative to the control group (P < 0.05). At the same TP concentration, the apoptosis rate of the free TP group was higher than that of TP-Exos group after 24 and 48 h (P < 0.05). The above experimental results suggest that TP-Exos can induce apoptosis of SKOV3 cells, but the effect is weaker in comparison with free TP, which was consistent with the results of MTT assay.

Both SK-Exos and free TP could induce apoptosis, but TP-Exos–induced apoptosis is less extensive than that caused by free TP. Encapsulation into exosomes may delay the cytotoxicity of TP and then weaken its ability to induce apoptosis in vitro. However, the delayed cytotoxicity of TP in TP-Exos may be related to the time required for TP-Exos to release TP. In this study, 10 ng/ml TP blocked cells in G0/G1 phase after 24 h, but 10 ng/ml TP-Exos blocked cells in G0/G1 phase after 48 h. Therefore, the time of apoptosis induced by TP-Exos may be 24 hours later than that of free TP.

### 3.6. Tumor Targeting of SK-Exos In Vivo

**Figure 6:**
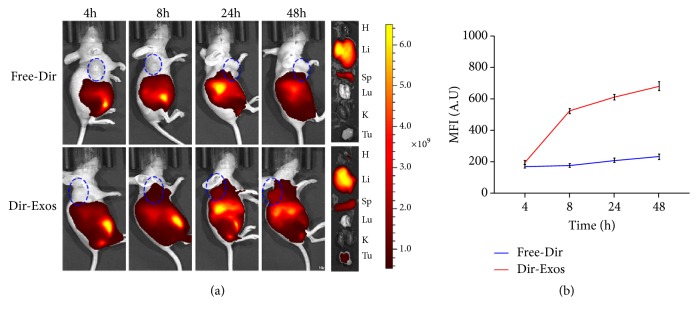
(a): Fluorescent images of SKOV3-tumor-bearing mice at 4, 8, 24, and 48 h postinjection of Free-Dir and Dir-Exos and fluorescent images of major organs (H: heart; Li: liver; Sp: spleen; Lu: lung; K: kidney; Tu: tumor). The blue circle is where the tumor is. (b) Statistical diagram of fluorescence intensity in tumor. Data are presented as mean ± SD (n =3).

To establish whether exosomes can be used effectively for drug delivery, understanding of their tumor targeting and distribution in vivo are important. It has been proved that intraperitoneal injection increased accumulation of drugs in tumor compared with intravenous injection [24]. Therefore, intraperitoneal injection has been used in this study. As shown in Figure 6, Dir was mainly distributed in the liver and spleen, and the fluorescent intensity in the tumor for Dir-Exos group increased with time, while that for Free-Dir group did not significant increase. The results demonstrate that exosomes can be rapidly uptaken by the liver and spleen, which is consistent with previous studies on the biodistribution of exosomes [25, 48–50]. We found that Dir-Exos could be recruited to the tumour site, which was consistent with the results of Ke Tang et al. [41], suggesting that SK-Exos may transport drugs to tumors via the circulatory system.

### 3.7. The Effect of TP-Exos on Ovarian Cancer In Vivo

**Figure 7:**
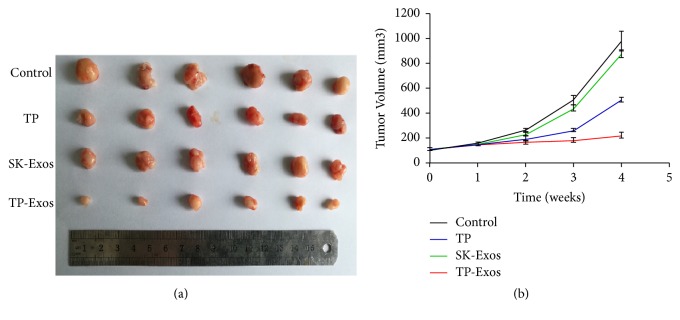
In vivo antitumor effect of the different drugs in SKOV3-tumor-bearing mice. (a) Photograph of tumor dissected from the mice after the last treatment. (b) The change of relative tumor volume (V/V initial) with treatment time. Data are presented as mean ± SD (n =6).

The antitumor efficacy of SK-Exos, free TP, and TP-Exos was evaluated based on the tumor volumes of the SKOV3 tumor-bearing mice. As shown in Figure 7, after 4 weeks of treatment, the tumor volume of TP-Exos group, control group, SK-Exos group, and free TP group was 222.51 ± 23.41 mm^3^, 977.35 ± 81.62 mm^3^, 877.59 ± 30.25 mm^3^, and 508.79 ± 17.96 mm^3^, respectively. These results suggested that tumor growth was inhibited in SK-Exos, free TP, and TP-Exos group compared with control group, and tumor suppression of TP-Exos group was significantly better than that of free TP groups.

The cytotoxicity of TP-Exos group is weaker than that of free TP group in vitro, but its antitumor effect in vivo is superior to free TP group. Exosomes derived from tumor cells carried tumor specific antigen, proteins, and miRNA, which could activate immune cells in vivo to play a synergistic antitumor effect. In addition, triptolide could significant increase the cell populations of T cells, B cells, monocytes, and macrophage. Furthermore, triptolide also could increase the phagocytosis of macrophage from peripheral blood mononuclear cells [51].

**Figure 8:**
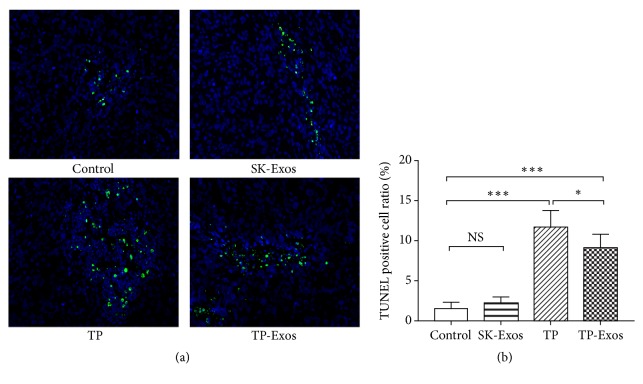
(a) Fluorescence image of TUNEL assay of the tumor tissue after treatment of different drugs. (b) Statistical analysis of TUNEL- positive cell ratio based on panel (a). Original magnification, 200×. Data are presented as mean ± SD (n = 6). NS means p > 0.05; *∗*p < 0.05 and *∗∗∗*p < 0.001.

The effect of TP-Exos on apoptosis of cells in transplanted tumors was examined by the TUNEL staining assay. As seen in Figure 8, the apoptotic cells displaying green fluorescence were found. Except for SK-Exos group, TUNEL-positive cells ratio in free TP group and TP-Exos group were higher than that in control group (p < 0.001). In addition, TUNEL-positive cells ratio in free TP group was higher than that in TP-Exos group (p < 0.05), which suggested that TP-Exos can induce apoptosis of cells in transplanted tumors, but the effect is weaker in comparison with free TP. The effect of TP-Exos on the proliferation and apoptosis of cells in vivo was consistent with that in vitro.

### 3.8. Toxicity of TP-Exos In Vivo

**Figure 9:**
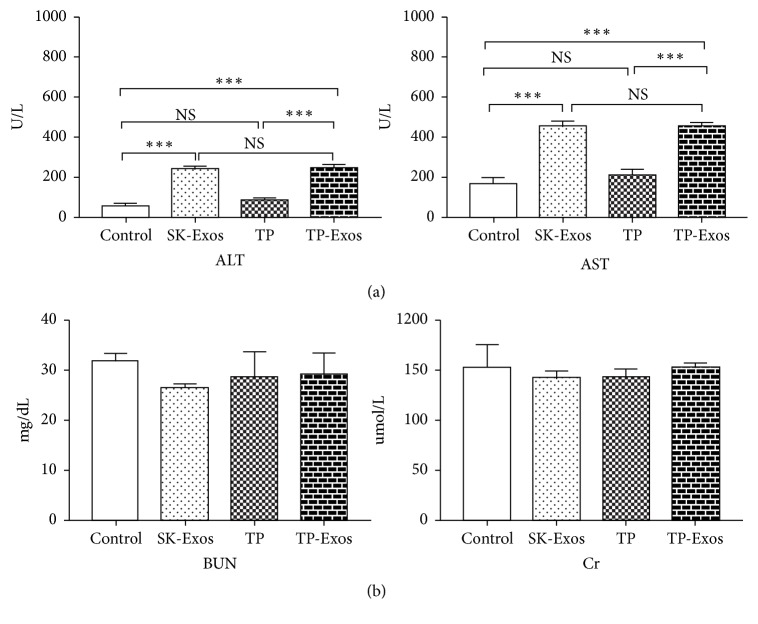
(a) Serum levels of ALT, AST in liver function. (b) Serum levels of BUN and Cr in renal function. Data is presented as mean ± SD (n = 3). NS means P > 0.05; *∗∗∗*P < 0.001.

In this study, the effect of TP-Exos on liver and kidney function was evaluated by detecting the level of AST, ALT, BUN, and Cr in serum of mice. As shown in Figure 9, compared with the control group, SK-Exos, free TP and TP-Exos groups had no effect on the level of BUN and Cr (p >0.05). Except for free TP group, the levels of AST and ALT in SK-Exos and TP-Exos groups were higher than that in control group (p < 0.001). In addition, the level of AST and ALT in TP-Exos group was not different from that in SK-Exos group (p >0.05) but significantly higher than that in TP group (p < 0.001).

**Figure 10:**
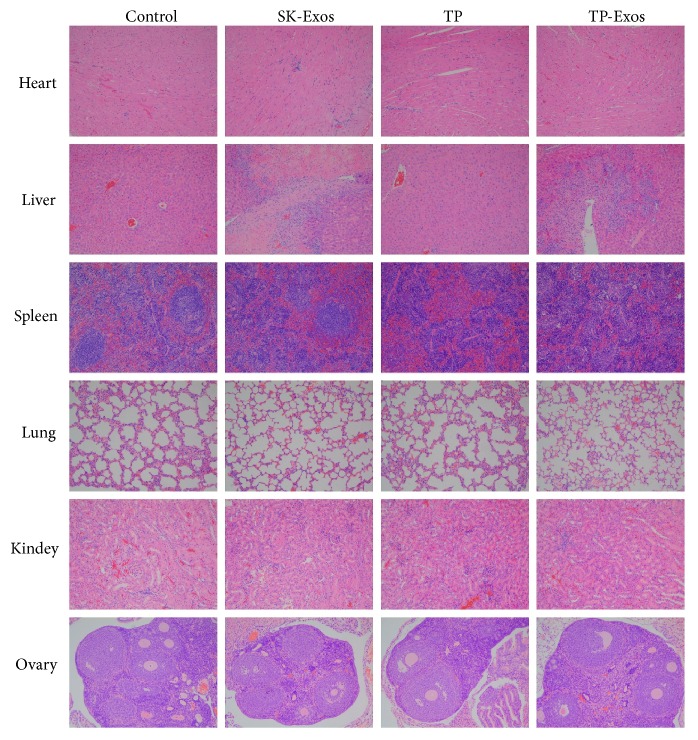
Histopathology of heart, liver, spleen, lung, kidney and ovary from mice after treatment. Original magnification, 200×.

The toxicity of TP-Exos to major organs was detected by histopathology in this study. As shown in Figure 10, no significant pathological changes were found in the heart, lung, kidney, and ovary in SK-Exos, free TP, and TP-Exos groups. There were obvious areas of necrosis in liver tissue in SK-Exos and TP-Exos group and the area of the germinal center of spleen tissue was not obvious in free TP and TP-Exos group.

All the above results showed that TP-Exos had no effect on renal function but could lead to liver dysfunction. TP-Exos have no obvious pathological damage to heart, lung, kidney, and ovary but has pathological damage to liver and spleen. In addition, we found that SK-Exos in TP-Exos may be the cause of liver injury and TP in TP-Exos may be the cause of spleen injury, indicating that TP-Exos may need to be further optimized to solve the problem of liver and spleen injury.

## 4. Conclusions

In this study, based on exosomes derived from SKOV3 cells, we have developed and characterized a novel anticancer drug formulation, TP-Exos. Then, we examined the effects of TP-Exos on proliferation and apoptosis of SKOV3 cells in vitro and in vivo. The results showed that TP-Exos may attenuate the cytotoxicity and apoptosis-inducing capability of TP toward SKOV3 cells but enhance the inhibitory effect of TP on cell proliferation. However, we further studied the toxicity of TP-Exos in vivo, and the results displayed that TP-Exos has toxic effect on liver and spleen. It proposed that the TP-Exos may be a promising strategy for ovarian cancer, but it needs to be further optimized to attenuate the damage to liver and spleen.

## Data Availability

The data used to support the findings of this study are included within the article.

## Supplementary Materials

Supplementary 1
* Supplemental Figure 1* Comparison of TP-Exos collected by ultracentrifugation (TP-Exos-UC) and ultrafiltration centrifugation (TP-Exos-UF). (a) Averaged FTLA (Fibre to the Last Active) Concentration/Size for NTA experiment. (b) Size distribution of SK-Exos and TP-Exos measured by NTA. (c) Particle concentration of SK-Exos and TP-Exos measured by NTA. Data are presented as mean ± SD (n = 3). *∗∗∗*p < 0.001.

Supplementary 2
* Supplemental Figure 2.1*  The HPLC profiles of TP standard solution, SK-Exos solution, and TP-Exos solution.* Supplemental Figure 2.2*  Calibration curve of TP by HPLC.

Supplementary 3
* Supplemental Table 1* Results of encapsulation efficiency of TP-Exos.
