# Supplementary material for: The Effect of Triptolide-Loaded Exosomes on the Proliferation and Apoptosis of Human Ovarian Cancer SKOV3 Cells
Source: Biomed Res Int. 2019 May 21;2019:2595801. doi: 10.1155/2019/2595801 (PMC6556367; doi:10.1155/2019/2595801)
Supplement: Supplementary 1 — Supplemental Figure 1 Comparison of TP-Exos collected by ultracentrifugation (TP-Exos-UC) and ultrafiltration centrifugation (TP-Exos-UF). (a) Averaged FTLA (Fibre to the Last Active) Concentration/Size for NTA experiment. (b) Size distribution of SK-Exos and TP-Exos measured by NTA. (c) Particle concentration of SK-Exos and TP-Exos measured by NTA. Data are presented as mean ± SD (n = 3). ∗∗∗p < 0.001. [file 2595801.f1.pdf]

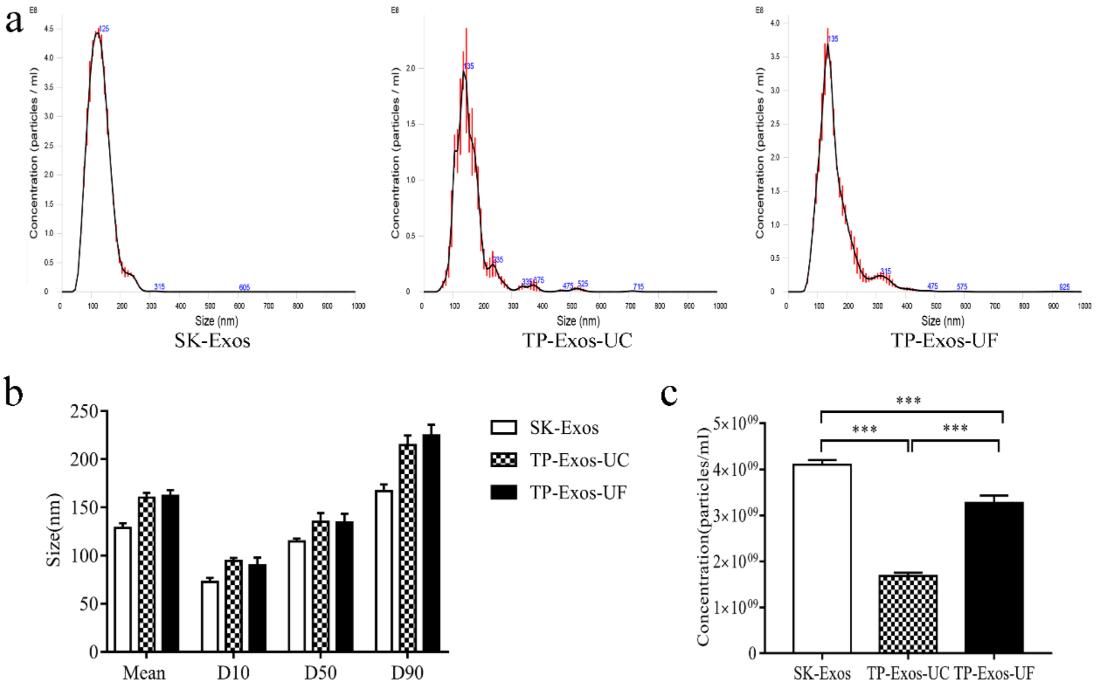


Supplemental Figure 1

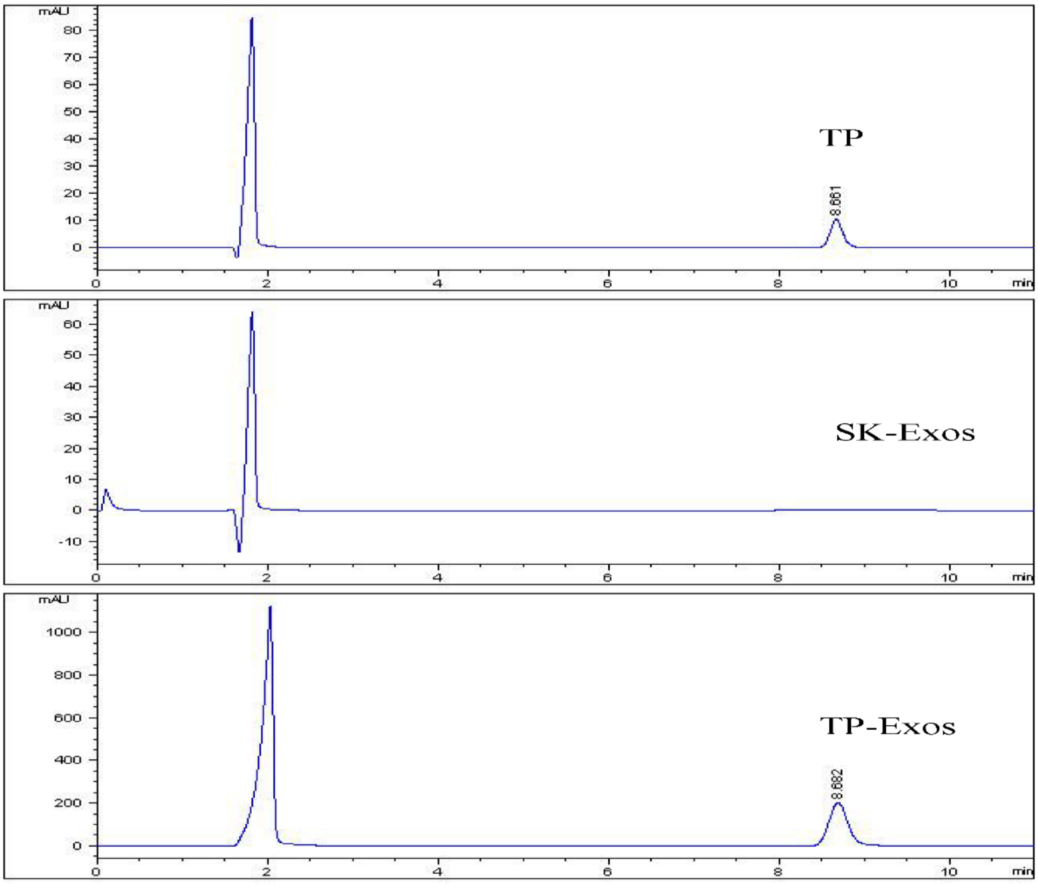


Supplemental Figure 2.1

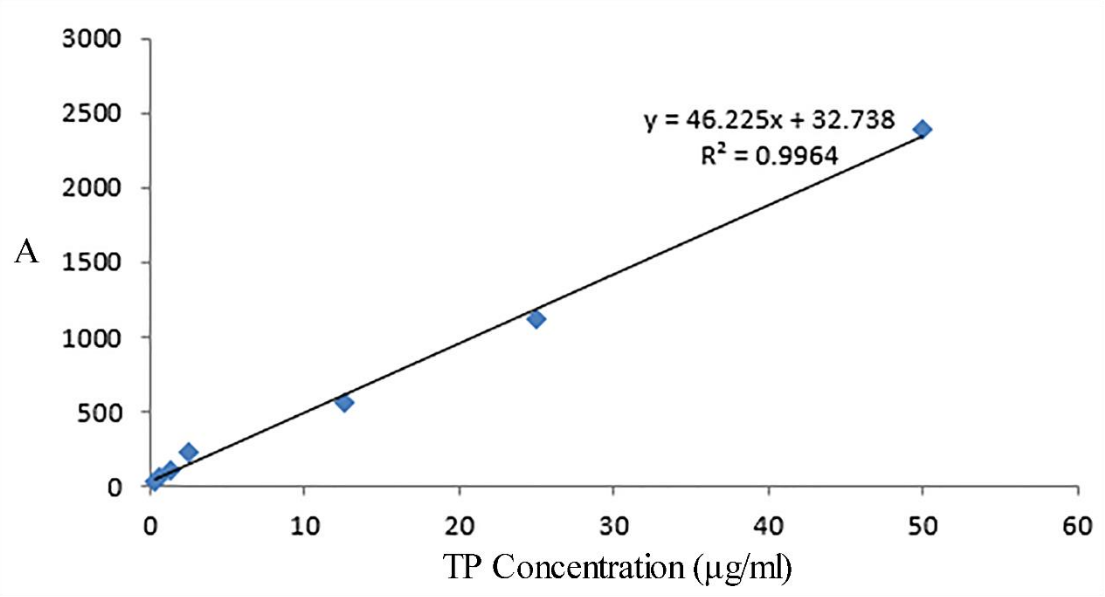


Supplemental Figure 2.2

Supplemental Table 1

| Sample | 1 | 2 | 3 |
|---|---|---|---|
| A | 118.9685 | 123.2367 | 120.7966 |
| Found (µg/ml) | 1.865 | 1.958 | 1.905 |
| Total amount of drug (µg) | 5 | 5 | 5 |
| Drug entrapped (µg) | 3.74 | 3.92 | 3.81 |
| Encapsulation efficiency (%) | 74.8 | 78.4 | 76.2 |
| Mean Encapsulation efficiency (%) | | 76.5 ± 1.8 | |
